# Enhanced glacial discharge from the eastern Antarctic Peninsula since the 1700s associated with a positive Southern Annular Mode

**DOI:** 10.1038/s41598-019-50897-4

**Published:** 2019-10-24

**Authors:** W. A. Dickens, G. Kuhn, M. J. Leng, A. G. C. Graham, J. A. Dowdeswell, M. P. Meredith, C.-D. Hillenbrand, D. A. Hodgson, S. J. Roberts, H. Sloane, J. A. Smith

**Affiliations:** 1 British Antarctic Survey, High Cross, Madingley Road, Cambridge, CB3 0ET UK; 2 Alfred-Wegener-Institut Helmholtz-Zentrum für Polar- und Meeresforschung, Bremerhaven, Germany; 3 NERC Isotope Geosciences Laboratory, British Geological Survey, Keyworth, Nottingham NG12 5GG UK; 4 College of Marine Science, University of South Florida, St. Petersburg, Florida USA; 5 Scott Polar Research Institute, University of Cambridge, Cambridge, CB2 1ER UK

**Keywords:** Cryospheric science, Palaeoclimate

## Abstract

The Antarctic Peninsula Ice Sheet is currently experiencing sustained and accelerating loss of ice. Determining when these changes were initiated and identifying the main drivers is hampered by the short instrumental record (1992 to present). Here we present a 6,250 year record of glacial discharge based on the oxygen isotope composition of diatoms (δ^18^O_diatom_) from a marine core located at the north-eastern tip of the Antarctic Peninsula. We find that glacial discharge - sourced primarily from ice shelf and iceberg melting along the eastern Antarctic Peninsula – remained largely stable between ~6,250 to 1,620 cal. yr BP, with a slight increase in variability until ~720 cal. yr. BP. An increasing trend in glacial discharge occurs after 550 cal. yr BP (A.D. 1400), reaching levels unprecedented during the past 6,250 years after 244 cal. yr BP (A.D. 1706). A marked acceleration in the rate of glacial discharge is also observed in the early part of twentieth century (after A.D. 1912). Enhanced glacial discharge, particularly after the 1700s is linked to a positive Southern Annular Mode (SAM). We argue that a positive SAM drove stronger westerly winds, atmospheric warming and surface ablation on the eastern Antarctic Peninsula whilst simultaneously entraining more warm water into the Weddell Gyre, potentially increasing melting on the undersides of ice shelves. A possible implication of our data is that ice shelves in this region have been thinning for at least ~300 years, potentially predisposing them to collapse under intensified anthropogenic warming.

## Introduction

The Antarctic Ice Sheet (AIS) is losing mass at an accelerating rate^1^, contributing to rising global sea-level^2^ and impacting ocean circulation through the injection of large volumes of freshwater^3,4^. Some of the most dramatic changes have been observed in the Antarctic Peninsula (AP) region, where up to 87% of glaciers are retreating^5^ and several ice shelves have collapsed^6,7^. At least some of the increased melting relates to rapid atmospheric warming associated with a more positive phase of the Southern Annular Mode (SAM)^8^, the leading mode of climatic variability in the Southern Hemisphere^9–11^. This has resulted in a strengthening of warmer westerly winds^12^ and increased surface melting on the eastern side of the AP^13^, which has been linked to the collapse of the Larsen B ice shelf^14^. Stronger westerlies also drive warm Circumpolar Deep Water (CDW) onto the western AP continental shelf, leading to enhanced transfer of heat to the underside of ice shelves^15^, and further melting. On the eastern AP, inter-annual fluctuations in bottom-water properties are governed by the wind-driven Weddell Sea Gyre, which is also sensitive to SAM^16,17^.

**Figure 1:**
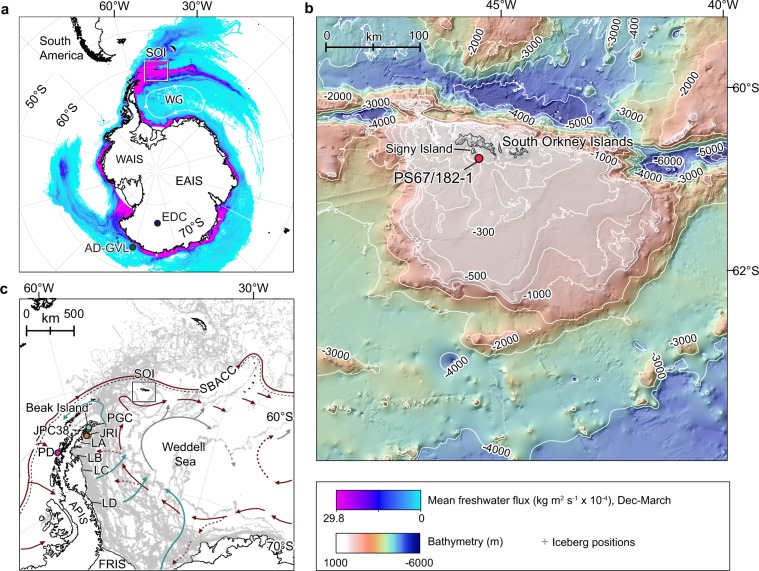
Location map. Core PS67/182-1 is located in the major pathways of glacier melt and icebergs discharging from Antarctica. (**a**) Modelled freshwater flux during summer (Dec-March) over the Southern Ocean in kg/m^2^/s (×10^−4^)^26^, visualized as a linear stretch between values using 0.2 standard deviations. The wind-driven, cyclonic Weddell Gyre (WG) is shown as a white line, South Orkney Islands (SOI) shown by the black square together with the locations of EPICA Dome C ice core (EDC) (blue circle) and the Adélie-George V Land δ^18^O_diatom_ record (AD-GVL; green circle)^21^. East Antarctic Ice Sheet (EAIS) and West Antarctic Ice Sheet (WAIS). (**b**) Shows location of core PS67/182-1 (red circle). Regional bathymetry from Dickens *et al*.^56^. (**c**) Shows the drift of icebergs (grey marker/shading) in the Weddell Sea embayment calving off Antarctic ice shelves from 1999 to 2009^28^ together with the trajectories of warm Circumpolar Deep Water (CDW, red - termed Warm Deep Water (WDW) in the Weddell Sea), Weddell Sea Bottom and Deep Water (WSBW/WSDW, light grey) and Ice shelf water (ISW, light blue)^71^. The southern boundary of the Antarctic Circumpolar Current (SBACC) is indicated by a black dashed line. Other key sites referred to in the text labelled; Palmer Deep (PD), Antarctic Peninsula Ice Sheet (APIS), Larsen A-D ice shelves (LA-D), James Ross Island ice core (JRI), Prince Gustav Channel (PGC) and Filchner-Ronne Ice Shelf (FRIS).

While the rates and drivers of modern warming are now well documented, the interplay between SAM and its impacts on ice sheet mass loss on longer, centennial to millennial timescales is poorly understood. This is principally a result of short instrumental records and a paucity of proxy data linking climate variability to glacier melting. Analysis of an ice core from James Ross Island (JRI) off the north-eastern tip of the AP (Fig. 1c) shows an intensification of summer melt layers since the fifteenth century (after A.D. 1400)^13^, which has been linked to atmospheric warming driven by a positive trend in SAM^18^. Although the relationship between atmospheric temperatures and melting is well-quantified for this high altitude (1,524 m) ice core site, there is little corresponding data on how the combined atmospheric and ocean temperature changes have driven retreat of glaciers and ice shelves at or close to sea level, where most of the recent mass loss has occurred^19^. One attempt to understand the thinning history of marine-terminating glaciers on the western AP analyzed the oxygen isotope composition of marine diatoms (δ^18^O_diatom_) in a sediment core from the near-coastal Palmer Deep^20^ (Fig. 1c). In this environment δ^18^O_diatom_ provides a proxy for glacier discharge^20–22^, derived from melting of floating glaciers/ice shelves and calved icebergs. This record revealed centennial to millennial-scale variations in glacial discharge during the past ~11,000 years with a trend to increasing discharge sometime during the late Holocene. However, the precise timing of this increase together with the driver(s) of change are uncertain due to insufficient resolution of this record, which is limited to four data points covering the past 500 years^20^. In addition to this, there is also a suggestion that the climate of the western and eastern AP was characterised by opposing temperature anomalies during the late Holocene^23^, further highlighting the need for additional constraints on glacier melting from a number of locations, covering a range of time-scales. Acquiring the data necessary to bridge the gap between the short instrumental record of mass loss (1992 to present^1^), estimates of surface melting in ice cores^13^ and existing marine records of glacial discharge from the western AP^20^ is the motivation of this study.

Here we present a high-resolution proxy record of glacier mass loss during the Holocene by measuring δ^18^O_diatom_ in marine core PS67/182-1 from the South Orkney shelf, at the northeastern tip of the AP (Fig. 1). Core site PS67/182-1 was targeted because it captures an integrated signature of glacial meltwater discharged from the Weddell Sea embayment – likely dominated by mass loss from the eastern AP (Fig. 1a,b)^24–27^ - rather than local glacier dynamics (see Supplementary Information). The restriction of melt to this specific geographic corridor of the ocean is steered by the strong cyclonic circulation of the Weddell Gyre (Fig. 1a), which controls not only the trajectory of icebergs (Fig. 1c)^27,28^, but also the cumulative flow of melt discharged from glaciers and ice shelves fringing the Weddell Sea embayment (Fig. 1a)^25,26^. In contrast, the South Orkney Islands (SOI) supports a small terrestrial ice cap, ~620 km^2^ in size. Onshore lacustrine sedimentation at nearby Signy Island (Fig. 1b) began over 7,000 years ago^29^ implying that the majority of deglacial ice melt after the Last Glacial Maximum (LGM; 26.5–19.0 kyr) had occurred by the early to mid-Holocene. Whilst moderate variations in glacier size have occurred since this time^30^, the cumulative flow of ice shelf and iceberg-derived meltwater to the region, as derived from a numerical model, is up to ~3000 mm/yr^31^ and thus nearly one order of magnitude greater than local meltwater input or precipitation (~400 mm/yr^32^). Our record thus provides a unique opportunity to assess the past sensitivity of ice shelves and glaciers in the NW Weddell Sea to known changes in climate.

## Results

### Variability in δ^18^O_diatom_ during the past 6,250 years

**Figure 2:**
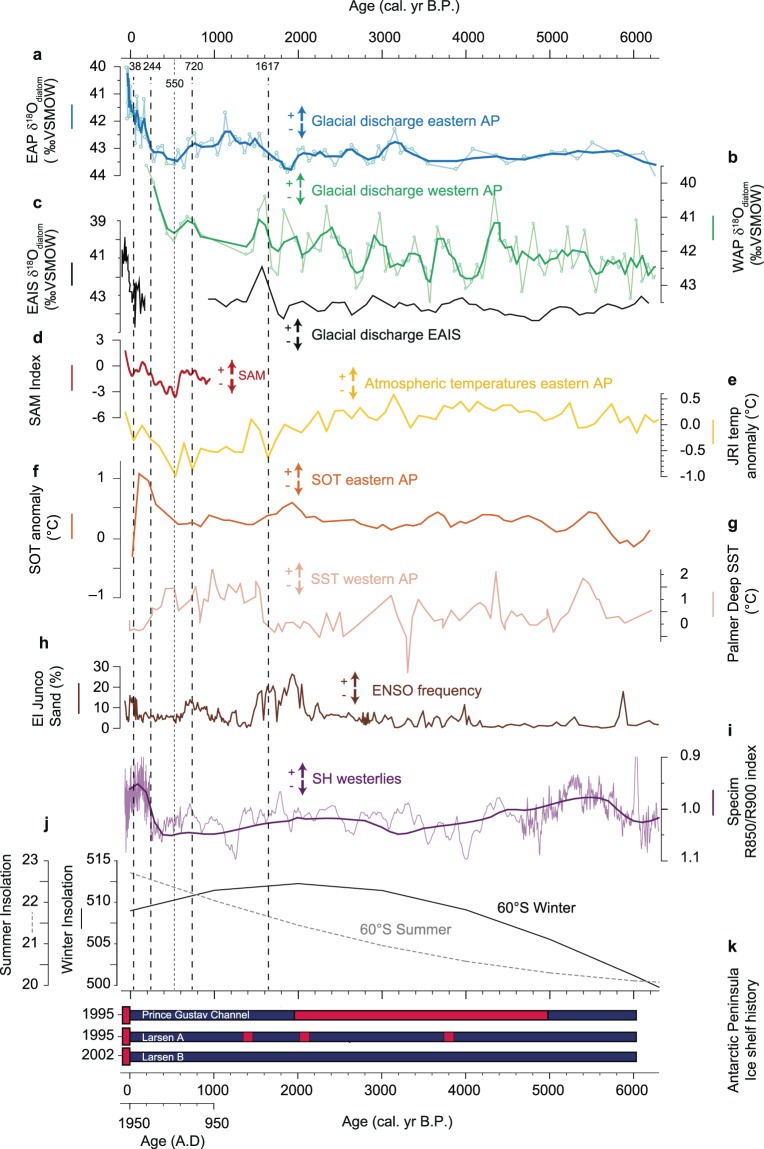
Holocene glacial discharge, climatic proxies and forcing mechanisms. (**a**) δ^18^O_diatom_ from core PS67/182-1 as a proxy for glacial melt with a 3 point moving average (blue; eastern Antarctic Peninsula (AP)). (**b**) δ^18^O_diatom_ derived glacial discharge record from Palmer Deep, ODP Site 1098 A with a 3 point moving average (green; western AP)^20^. (**c**) δ^18^O_diatom_ derived glacial discharge record from Adelie-George VI Land coast (black; EAIS glacial discharge)^21^. (**d**) SAM index with 70 year loess filter (red)^18^. (**e**) 100 year average deteurium reconstructed temperature anomalies from James Ross Island (JRI) ice core (yellow)^23^. (**f**) Subsurface ocean temperature (SOT) for eastern AP^38^. (**g**) TEX^86^_L_ SST derived temperature record from core Site 1098, Palmer Deep (peach)^39^. (**h**) El Junco Sand as an indicator of ENSO frequency (brown)^43^. (**i**) hyperspectral ratio (*R*850/*R*900) from a lake record on Macquarie Island (purple line), as proxy for Southern Hemisphere westerlies^50^. Lower values equate to stronger winds (thicker line is with 100-year interval second-order LOESS smoothing). (**j**) December and June insolation values at 60°S^72^. (**k**) Holocene reconstructions of the presence (dark blue bar) and absence (red bar) of AP ice shelves that have broken up or retreated significantly in the past few decades (modified from Hodgson^6^). Year of recent ice shelf collapses appears left of panel. All data are plotted against age (cal. yrs BP) with an additional A.D. age scale for the last 1000 years. Dashed/dotted vertical lines show significant change points in the δ^18^O_diatom_ data (see Materials and Methods).

Core PS67/182-1 recovered an 18-m sequence of laminated diatom-rich mud, deposited in a seasonally open marine environment (see Materials and Methods for more details). The sediment sequence was dated using both ^210^Pb and ^14^C methods, with age-depth modelling performed in CLAM v2.2^33^ (see Materials and Methods, Fig. S6). The base of PS67/182-1 is dated to 6,250 calibrated years before present (where ‘present’ corresponds to A.D. 1950). Change point analysis was undertaken to identify the timing of significant changes in δ^18^O_diatom_ data (see Materials and Methods, Fig. S7). The δ^18^O_diatom_ record shows relatively uniform values from 6,250 to 1,617 cal. yrs BP (Fig. 2a). The first significant change points in the data show an increase in glacial discharge (i.e., trend to lower δ^18^O_diatom_ values) between 1,617 and 720 cal. yr BP followed by a decline (Fig. 2a). Since c. 550 cal. yr BP (~A.D. 1,400) discharge has increased steadily with a significant change-point at 244 cal. yr BP (~A.D. 1706), where the magnitude of discharge emerges beyond the range of natural variability set during the past 6,250 years (Fig. 2a). A further significant change point at 38 cal. yr BP (A.D. 1912) (Fig. 2a) shows a marked acceleration in the rate of ice melt in the last 106 years. The apparent lack of correspondence between δ^18^O_diatom_ and ice-rafted debris (IRD) counts (>2 mm) and/or sand fraction (Fig. S4g) particularly throughout this period of elevated glacial discharge suggests that the δ^18^O_diatom_ signal at core site PS67/182-1 is mainly controlled by ice shelf, glacier and iceberg melting along the eastern AP rather than melting of icebergs directly over the core site. Other measured proxies also vary throughout the Holocene (Fig. S4) but there is no clear long-term relationship between the δ^18^O_diatom_ record and these proxies. Bulk productivity indicators (TOC, *b*Opal, Ba/Ti) show centennial to millennial-scale variability (Fig. S4a–c) that are broadly anti-correlated with terrigenous input (sand fraction, mean sortable silt (mSS) and magnetic susceptibility (MS)) suggesting that part of the productivity signal is probably related to dilution by terrigenous material. However, the lack of a clear relationship between TOC/*b*Opal and δ^18^O_diatom_ implies that the δ^18^O_diatom_ signal is unlikely to be influenced or overprinted by local productivity changes. Thus, and consistent with previous work, we are confident that lower δ^18^O_diatom_ values equate to increased glacial discharge.

### Drivers of glacial discharge during the past 6,250 years

#### 6.25–1.62 ka

The consistently high δ^18^O_diatom_ values prior to c. 1,617 cal. yr BP are indicative of a marine-water dominated signal with a reduced influence from glacier and iceberg melting relative to modern-day values (Fig. 2a). We attribute this period of low variability, relatively uniform glacial discharge to normal growth and calving of glaciers following retreat from LGM limits. Regional deglaciation was largely complete by the early Holocene in the Weddell Sea embayment^34,35^, and although local glacier-front variability has been observed in some areas^36^ the ice sheet appears to have reached a stable configuration by the early to mid-Holocene. Multi-proxy analysis of marine core JPC38 recovered at the northern end of Prince Gustav Channel (Fig. 1c) indicates that the eastern AP region experienced cooler conditions, glacier advance and sea-ice expansion between 5,000–1,900 cal. yrs BP^37^ while sub-surface ocean temperatures (SOT = 50–400 m water depth) shows centennial-scale warm events that are superimposed on a gradual warming trend (0.3 °C) from ~7,000 to 500 cal. yr BP (Fig. 2f)^38^. The JRI ice core shows an interval of stable climate persisted from, 9,200 to 2,500 yr BP^23^. Reduced glacial discharge^20^, increased sea-ice cover and a relatively cool sea surface temperatures (SST)^39^ were also observed on the western AP at this time, suggesting coherent temperature trends during the mid-Holocene (Fig. 2g). Cooler atmospheric conditions during this time interval have been linked to reduced solar insolation (Fig. 2j) which would have reduced glacier melt directly along both sides of the Peninsula. Barbara *et al*.^37^ further suggest that a northward displacement of the Antarctic Circumpolar Current (ACC) occurred at this time, reducing the entrainment of warm CDW into the Weddell Gyre resulting in sea-ice growth and reduced ice-shelf melting. The apparent increase in δ^18^O_diatom_ variability between c. 3,200 and 1,900 cal. yr BP is broadly coincident with a mid-Holocene climate optimum inferred from multiple indicators of increased biological production in lakes on nearby Beak Island (c. 3,800–1,400 cal. yr)^40^ and on Signy Island (3,169 and 2,120 cal. yr BP)^41^. (Fig. 1c). It also corresponds with a possible, though poorly dated, period of ice-shelf absence in Prince Gustav Channel^42^ (Fig. 2k).

#### 1.62–0.72 ka

Glacial discharge is more variable between 1,617 and 720 cal. yr BP (A.D. 333–1230) peaking around ~1,100 cal. yr BP before declining (Fig. 2a). This variability, however, is not identified in our change point analysis. Climate variability in the Peninsula region during this interval has been linked to a peak in summer insolation and an intensification of El Niño/Southern Oscillation (ENSO)^43,44^ (Fig. 2h). Along the western AP, the high SSTs between 1,600 and 500 cal. yr BP (Fig. 2g) have been attributed to La Niña events^39^, which are thought to have brought warm air from lower latitudes towards Antarctica via the westerlies^12^ and driven increased frontal melting of marine-terminating glaciers^20^. The lack of distinct correlation between glacial discharge at site PS67/182-1 and ENSO-proxies (Fig. 2a,h) throughout this interval suggests that ice masses on the eastern AP and Weddell Sea embayment responded non-linearly to climate forcing, or that the amplitude of forcing was insufficient to drive the large-scale increases in glacial discharge that is seen during subsequent stages (i.e., the last ~500 years). In this context, Mulvaney *et al*.^23^ have noted the development of opposing east-west temperature trends during the late Holocene (~1,500–550 cal. yrs BP), where warmer SST on the western AP coincides with cooler atmospheric temperatures on the eastern side. Opposing temperatures during this period have been related to the establishment of Antarctic dipole-like conditions, driven by opposite temperature and sea-ice anomalies in the Weddell and Amundsen-Bellingshausen seas. The development of dipole-like conditions during the late Holocene, which has been linked to ENSO, might explain why the long-term trend to increasing glacial discharge on the western AP, starting around ~2,500 cal. yr BP^20^ (Fig. 2b), is not replicated at site PS67/182–1. Limited ocean-driven melting is also supported by cooler, or at least stable, SOT inferred from core JPC38 (Fig. 2f)^38^. Recently, Charman *et al*.^45^ have argued against opposing temperature trends based on the analysis of moss banks on either side of the Peninsula. Either this suggests an incomplete understanding of west-east AP climate trends during this time period, or that individual proxies from marine/terrestrial archives are sensitive to different, or even multiple, climate variables.

#### 0.72 to 0 ka

**Figure 3:**
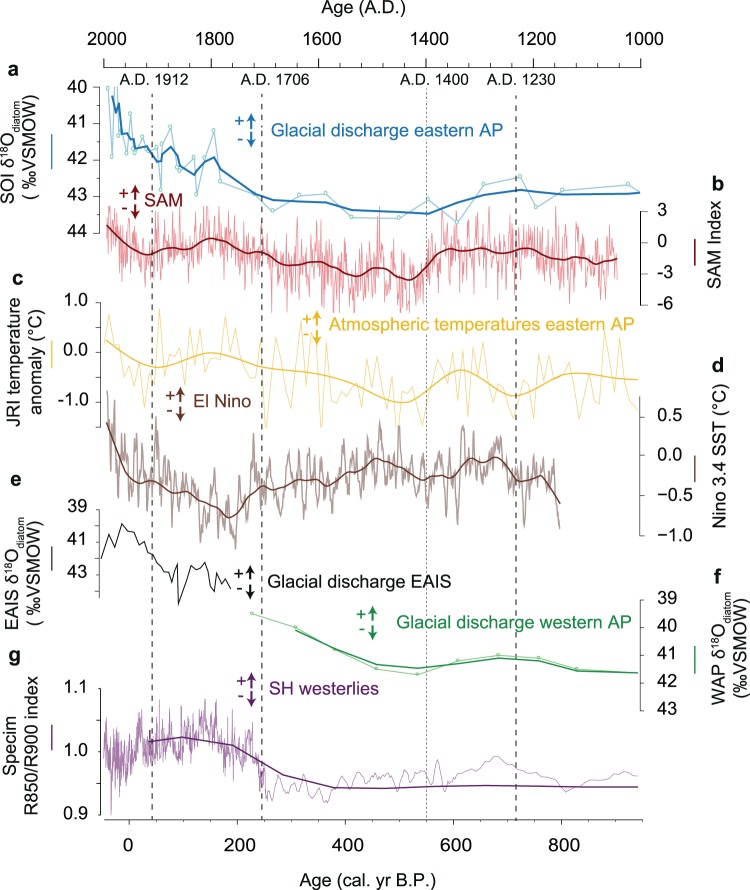
Climate evolution during the past 1000 years. (**a**) Glacial melt from the δ^18^O_diatom_ index of PS67/182-1 with a 3 point moving average (blue). (**b**) The Southern Annular Mode (SAM) with a 70 year loess filter (red)^18^. (**c**) Temperature anomalies from JRI ice core with a 3 point moving average (yellow)^23^. (**d**) Multiproxy reconstruction of Niño3.4 sea surface temperature (SST) with a 3 point moving average (dark red line)^73^. (**e**) δ^18^O_diatom_ derived glacial discharge record from Adelie-George VI Land coast (black)^21^. (**f**) δ^18^O_diatom_ derived glacial discharge record from Palmer Deep, ODP Site 1098 A with a 3 point moving average (green)^20^. (**g**) hyperspectral ratio (*R*850/*R*900) from a lake record on Macquarie Island (purple), as proxy for Southern Hemisphere westerlies^50^. Lower values equate to stronger winds (thicker line is with 100-year interval second-order LOESS smoothing). The upper part of this record (A.D. 1903 to present; denoted by a black line) is potentially disturbed by erosion associated with rabbit activity^50^. Dashed/dotted vertical lines show significant change points in the δ^18^O_diatom_ data (see Materials and Methods).

Change points at 720 cal. yr BP (A.D. 1230) and 244 cal. yr BP (A.D. 1706) bracket pronounced changes in discharge at site PS67/182-1, which first declines until ~550 cal. yr BP (A.D. 1400) before displaying a pronounced increasing trend until the modern day (A.D. 2005). At the same time, both the JRI ice core^23^ and the Beak Island lake record^40^ on the eastern AP show a warming trend from 550 and 543 cal. yrs BP respectively (Fig. 2e), while SOTs on the eastern AP appear to increase from ~500 cal. yr BP (Fig. 2f)^38^. Glacial discharge on western AP also shows a pronounced increase around 550 cal. yr BP, although this is limited to 4 data points and is also superimposed on a longer-term increasing trend that started much earlier (~2,500 cal. yr BP)^20^ (Fig. 2b). Warmer atmospheric temperatures on the eastern AP after ~550 cal. yr BP have been associated with variations in SAM which shows a significant switch - from its positive to negative phase between 1300 and 1460 cal. yr BP - followed by a trend to positive SAM since the mid-fifteenth century^18,40^ (Fig. 2d). We note that variations in glacial discharge at site PS67/182-1 also appear to follow steps in the SAM, with low but increasing discharge from ~550 cal. yr BP (A.D. 1400) until 244 cal. yr BP (A.D. 1706) associated with a predominantly –SAM, but increasing to its positive phase thereafter (Fig. 3a,b). After 244 cal. yr BP (A.D. 1706), glacial discharge increases rapidly to a level that is unprecedented during the past 6,250 years, coincident with predominantly + SAM as well as increasing El Niño conditions (Figs 2a, 3 and S2). We therefore argue that + SAM acted as an important driver of enhanced glacial discharge along the eastern AP, particularly after A.D. 1706.

### SAM as a driver of glacial discharge during the late Holocene

SAM denotes the pressure gradient between mid and high latitudes^8^ and, during a more positive SAM, the belt of westerlies intensifies and contracts poleward, bringing stronger, warmer westerlies to the AP region. Analysis of contemporary temperature data (spanning 1979–2015) reveals that SAM impacts the west and eastern sides of the AP in different ways^12^. On the eastern AP, inter-annual variability of temperatures is most sensitive to zonal wind (west-east) anomalies crossing the Peninsula and resultant leeside adiabatic warming (Föhn winds) rather than to meridional wind anomalies (i.e., north-south variations), which is closely tied to variability in the zonal portion of the SAM pattern^12^. More frequent Föhn events drive enhanced surface melting that has been linked to collapse of the Larsen B Ice Shelf in 2002 through hydrofracture^14,46^. The oceanic response to the shifting SAM – on the eastern side of the AP - is mostly through Ekman transport^16^. Positive SAM is associated with increased wind stress and reduced sea-ice^47,48^ and, over longer time periods, stronger westerlies promote greater entrainment of warm CDW water into the Weddell Gyre^16^ with the potential to increase melting of fringing ice shelves^38^.

We hypothesise that a progressive shift to positive SAM since the mid-fifteenth century, and particularly since the early 1700s promoted stronger westerlies that triggered increases in glacial discharge, via the mechanism that we see today; that is, atmospheric and ocean-driven thinning and retreat of ice shelves. Whilst there is a dearth of wind-proxies for the AP covering this time-interval, sea salt aerosol (ssNa+) concentrations in EPICA Dome C ice core^49^ (Figs S1 and S2) indicate increasing and decreasing wind in phase with SAM from ~500 cal. yr BP (Fig. S2). In addition, wind proxy records from sub-Antarctic Macquarie Island^50^ (Fig. 3g) and southernmost Patagonia^51^ suggest a significant poleward displacement and likely intensification of westerlies ~A.D. 1700, when glacial discharge at site PS67/182-1 increases beyond the long-term mean (Fig. 3). At the same time, atmospheric and ocean warming on the eastern AP has been observed from ~550–500 cal. yr BP, increasing in progressive steps towards the present day (Fig. 2e,f)^23,38^. The apparent down turn in SOT around A.D. 2000 (Fig. 2f) has been described in terms of intensified downwelling linked to the well-publicized phase of atmospheric cooling of the Peninsula region^38^. Although speculative, we also note that enhanced glacial discharge has been observed along the Adélie-George V Land coast (Figs 1c and 3e) from ~A.D. 1700^21^ which could imply a circum-Antarctic response to SAM/wind forcing. Our explanation is consistent with observations linking + SAM with the recent retreat of outlet glaciers along the Pacific coastline of EAIS, either through rising air temperatures or increased coastal upwelling of CDW^52^, although is at odds with the original interpretation linking the decrease in δ^18^O_diatom_ to intensified ENSO^21^. Irrespective of the driver, it would appear that at least three sectors of the ice sheet (western and eastern APIS, EAIS) have undergone enhanced mass loss during the past ~300–500 years. In the case of the eastern AP, the most recent changes (since A.D. 1706) are unprecedented during the past 6,250 years.

The mechanisms driving SAM trends during the last millennia are only partially understood. Coupled climate simulations indicate that increasing solar irradiance after the Spörer Minimum (~A.D. 1415–1534) resulted in a small positive forcing on SAM^10,18^. Abram *et al*.^18^ also note a strong co-dependence between ENSO and SAM during the last 1000 years, suggesting the maximum in Niño3.4 SST during the fifteenth century might have contributed to the SAM minimum (Fig. 3b,d). Instrumental studies indicate that ENSO variability in the tropical Pacific interacts with storm tracks in the south Pacific via a Rossby wave train, whereby El Niño (La Niña) events drive cool (warm) conditions on the AP and are associated with negative (positive) SAM states^53^. This is seen as an inverse relationship between the Niño3.4 reconstruction and the JRI temperature record, lending support to the idea that El Niño and negative SAM was a persistent feature during the late Holocene^18^. El Niño is thought to become a secondary influence on SAM during the twentieth century when a positive trend in SSTs (El Niño) (Fig. 3d) would be expected to impose negative forcing on the mean state of SAM^18^. However, SAM shows a continuing trend to a more positive state (manifest as greater glacial discharge at our site) which is thought to reflect the dominant influence of rising greenhouse gas levels and ozone depletion. However, despite evidence for a clear co-dependence between SAM and ENSO during the late Holocene, questions exists about the relative impacts on AP temperatures, and how this might vary on the west and east sides of the Peninsula.

### Summary and outlook

We have shown that glacial discharge along the eastern AP in the NW Weddell Sea embayment has varied throughout the past 6,250 years (Fig. 2a). Initially long-wavelength mechanisms such as orbital forcing and ENSO frequency provide plausible mechanisms for these changes. An increasing trend in glacial discharge is seen from ~A.D. 1400 (550 cal. yr BP), although it wasn’t until A.D. 1706 (244 cal. yrs BP) that discharge at site PS67/182-1 exceeded the level experienced during the past 6,250 years. Comparison with available proxy data indicates that increases in glacial discharge appear to have been driven, at least in part, by a positive phase of the SAM and intensification of westerly winds (Figs 3 and S2), acting on both the atmosphere and ocean to drive thinning of fringing ice shelves and marine-terminating glaciers. If our interpretation is correct, one possible implication of our data is that the early onset of melting (after A.D. 1706) has contributed to the near synchronous loss of ice shelves along the eastern AP^6,7^ - most notably the Larsen A-Prince Gustav Channel in 1995 and Larsen B in 2002 (Fig. 2k) - providing further support to the idea that ice shelves on the eastern AP have been predisposed to collapse by hundreds, and in some cases thousands^54^ of years of thinning. This contrasts with the irregular timing of Holocene ice shelf collapses known to date (Fig. 2k). Indeed, no single forcing mechanism has been identified as providing a tipping point for past ice shelf retreat^6^ which could explain why the signal of past collapses in our δ^18^O_diatom_ record is more diffuse than the pronounced changes witnessed in recent decades.

Models predict further poleward intensification of the westerlies^11,18^ and continued greenhouse warming around Antarctica^55^. Given the sensitivity of the APIS (and Antarctic Ice Sheet more generally) to variations in the westerlies, it is likely that mass loss will continue to accelerate through the mid-late twenty first-century, raising global sea-level^1^, impacting marine ecosystems^3^ and potentially leading to meltwater-induced sub-surface ocean warming which has the potential to drive even greater melting underneath ice shelves^4^.

## Materials and Methods

### Core location and material

Piston core (PC) PS67/182-1 (Lat. −60.792700, Long. −45.494300) and corresponding trigger core (TC) were recovered from 364 m water depth within the eastern tributary of Signy Trough, ~15 km south of Coronation Island, South Orkney Islands (SOI) (Fig. 1a,b), during cruise ANT-XXII/4 (PS67) on the RV *Polarstern*. Core PS67/182-1 penetrated a thick (>60 m) package of acoustically laminated sediments (Fig. S3c), interpreted as a hemipelagic postglacial sediment drape^56^. The 18-m PC consists of 5Y 4/3 to 5Y 4/4 olive, bioturbated and weakly stratified diatomaceous mud (Fig. S3a,b) with isolated dropstones and low sand concentrations (Fig. S4g). X-ray fluoresce (XRF) area counts of two elements, Silica (Si) and Titanium (Ti), were employed to splice the PC (Fig. S5, blue line) and TC (Fig. S5, red line). Plotted on the same vertical scales, with no horizontal (depth) offset, both elements exhibit a strong correlation indicating no significant offset or surface sediment loss in the PC.

The South Orkney Islands (SOI) are located at the north-western limb of the cyclonic flowing Weddell gyre (Fig. 1a), which is the dominant circulation feature south of the Antarctic Circumpolar Current (ACC) in the Southern Ocean’s Atlantic sector^57^. Water flows westwards in the southern limb (Weddell Sea embayment) and eastwards in the northern limb of the gyre (Fig. 1c). Due to its divergent nature, Ekman pumping causes major upward transport of sub-surface water in the gyre’s interior^16^. Formation of deep and bottom waters occurs in the southern and western parts of the gyre^58^; dense water originating here is found in most of the Southern Hemisphere as Antarctic Bottom Water. Surface water of the gyre is exchanged with the adjacent ACC along all of its northern and eastern boundaries (Fig. 1c). Warm Circumpolar Deep Water (CDW), locally termed Warm Deep Water (WDW), enters the Weddell gyre east of the Greenwich Meridian and cools as it traverses westward within the coastal current forming the gyre’s poleward limb. It eventually reaches the southwestern Weddell Sea, where it is modified to Modified Warm Deep Water (MWDW), and then spreads onto the eastern shelf (Fig. 1). MWDW is converted by wintertime sea-ice production into high-salinity shelf water (HSSW). The HSSW that travels to the deep grounding lines in the south of the ice shelf and supplies heat for melting, generates the ice shelf water (ISW)^59^. The export of the dense shelf water across the outer edge of the shelf, leads to Weddell Sea Bottom Water (WSBW). Seasonal fluctuations of WSBW properties are governed by the seasonal cycle of the winds over the western margin of the Weddell Sea. Inter-annual fluctuations are linked to the variability of the wind-driven Weddell Sea gyre and hence to large-scale climate phenomena such as the SAM and El Niño/Southern Oscillation^16^.

### Core processing and analyses

Volume specific magnetic susceptibility (MS) and wet-bulk density (WBD) were analysed at 1 cm intervals on whole cores using a GEOTEK multi-sensor core logger (MSCL; fitted with a Bartington MS2 susceptibility meter with a MS2C loop sensor of diameter 14 cm) at the Alfred Wegener Institute for Polar and Marine Research (AWI) (Bremerhaven, Germany). MS was also measured on split cores at 1 cm intervals (using a Bartington MS2 susceptibility meter with MS2F sensor). Lithology, colour and sedimentary structures were described visually on the split cores, and refined using X-radiographs of 1 cm-thick sediment slabs taken parallel to the core axis. Water contents were determined by weighing samples of 10 cm^3^ volume before and after freeze drying. Grain-size composition (gravel-sand-mud) was analysed on samples of ~60 cm^3^ volume from 1-cm intervals by wet- and dry sieving over >63 μm and 2 mm, respectively. The mud fraction (<63 μm) was retained and used to analyse sortable silt (SS) following McCave *et al*.^60^. Biogenic opal was first digested using a 2 molar Sodium Hydroxide (NaOH) solution, heated at 90 °C for an hour. The NaOH solution was removed with three deionised water rinses. In addition, organic material was digested using a 30% H_2_O_2_ solution, shaken for 24 hours at room temperature. Samples were placed on a vertical carousel for 24 hours prior to analysis and the sonicator was used for 30 seconds before each measurement. Analyses was performed on a Coulter Counter (Multisizer-3) at the Earth Sciences Department, University of Cambridge and mean SS calculated using the equations in McCave *et al*.^60^. Carbonate was not removed as it forms a very small component of the samples. Ice-rafted debris (IRD; >2 mm in size) was counted at 2 cm intervals in PS67/182-1 using high-resolution X-radiographs^61^. Total organic carbon (TOC) was measured every 10 cm with an Elementar Vario EL at the AWI. TOC contents were determined after removal of the total inorganic carbon (TIC, carbonates) with HCl using an ELTRA CS-2000. Biogenic opal (*b*Opal) was analysed at a sampling interval of 10 cm using an automated leaching technique^62^. The archive halves of the sediment cores were scanned with an Avaatech XRF-core scanner^63^ at AWI with a sampling interval of 1 cm and spot size of 1 cm^2^.

### Age-model

A chronology for the sediment sequence was established by AMS radiocarbon (^14^C) dating of calcareous micro-fossils and the humic acid in the organic fraction of the sediments where no microfossils were present (Table S3) in combination with ^210^Pb profile for PS67/182-1 TC. Microfossils were hand-picked from the 63 µm-2 mm fraction and submitted to Scottish Universities Environmental Research Centre (SUERC), Beta Analytic, Florida (BETA), University of Kiel (KIA) and Queen’s University Belfast (UBA) for accelerator mass spectrometry (AMS) radiocarbon dating. The ^210^Pb and ^137^Cs activities were measured on 1–2 cm thick sediment slices by gamma spectrometry using a Canberra ultra-low background Ge-detector. Specifically, ^210^Pb was measured by way of its gamma peak at 46.5 keV, ^226^Ra by way of its granddaughter ^214^Pb (peaks at 295 keV and 352 keV), and ^137^Cs by way of its peak at 661 keV. The TC showed surface contents of unsupported ^210^Pb of 392.73 Bq kg^−1^ with a tendency for exponential decline with depth in the upper ~20 cm (Fig. S6b). ^137^Cs was very low and typically below detection levels. Ages were determined using the constant rate of supply (CRS) model with errors calculated on the basis of error propagation^64^. All ^14^C samples were calibrated using the Marine13 calibration curve^65^ within CLAM v2.2^33^. Because the marine reservoir is poorly constrained in this region^66^, we follow the approach used by the RAISED Consortium^35^ and apply uniform marine reservoir of 1,300 years (ΔR of 900 years). All ages are reported as calibrated years before present (cal. yr B.P.), where ‘present’ corresponds to A.D. 1950. The calibrated ages exhibit a near linear increase with depth (r^2^ = 0.9891 with linear fit) with no age reversals (Fig. S6a,c and Table S3). The two lowermost humic acid ages (1202 and 1841 cm) show a close agreement with the nearest carbonate ages (Fig. S6b and Table S3). Down-core excess in the ^210^Pb profile for PS67/182-1 TC (Fig. S6b), with high activity at the surface and a down-core profile showing constant decay, suggests that the TC recovered the modern surface. Based on the TC/PC splice (Fig. S5), it is assumed that the surface sediments of the PC also reflect modern deposition. Thus, it is likely that the humic acid sample at 1.5 cm, which yielded a calibrated age of 440 years is unreliable, either because of contamination from fossil carbon or post depositional alteration. When constructing the age-depth model this humic acid age was removed and replaced with the ^210^Pb data (0 cm = A.D. 2005 or −55 cal. yr B.P.). Age-depth modelling of the ^210^Pb and ^14^C age-data was undertaken with CLAM v2.2^33^ using linear interpolation between neighboring levels (Fig. S6a). Ages were calculated every 0.5 cm. Interpolated ages in the text are based on the ‘best-fit’ age from the CLAM age-depth model. All median calibrated ages and ranges quoted are 2σ error (Table S3). Error is typically below ± 10 yr during the past ~180 years, and between ± 10 and ± 120 years for the remainder of the Holocene (Table S1).

### Change point analysis

Standard mean change point (CP) analysis^67^ was calculated to determine the periods when significant trend changes occurred in the δ^18^O_diatom_ dataset (Fig. S7). Analysis was performed on 10 (Fig. S7a) and 100 year (Fig. S7b) LOESS-filtered (2^nd^ order poly smoothing) datasets, which is most suitable for identifying changes throughout the 20^th^ Century and Holocene, respectively. Significant CPs were identified at 38 cal. yr BP, 244 cal. yr BP; 720 cal. yr BP and 1,617 cal yr BP (Figs S7a,b and S2, S4 vertical dashed lines). Both the 100 year and 10 year LOESS filtered datasets yield consistent CPs, although the 20^th^ century CP (38 cal. yr BP) is missed in the 100-yr smoothing (Fig. S7b).

### δ^18^O_diatom_ analysis

Samples were prepared following the methods outlined in Swann *et al*.^68^. ~0.5 g samples were digested using a 30% Hydrogen Peroxide (H_2_O_2_) solution, heated to ~70 °C for up to two weeks. The peroxide solution was washed off with three deionised water rinses in the centrifuge. Carbonate was removed using a 5% Hydrochloric (HCl) acid solution, left at room temperature for at least 24 hours. The HCl solution was also washed off with three deionised water rinses. The diatoms were isolated from the terrigenous material using the heavy liquid Sodium Polytungstate (SPT), made up to an initial specific gravity of 2.25. Once the cleaned diatom residues were obtained, they were sieved through a 38 μm sieve, and filtered through a 3 μm membrane to further remove contaminants (e.g., sponge spicules). This step removes the vast majority of whole spicules although occasional spines may pass through the sieve. Sample purity was assessed on a sub-set of samples prior to analysis using X-Ray Fluorescence (XRF) (Table S2); an aluminium concentration (proxy for terrigenous material) below 1% is a useful threshold for assessing the degree of terrigenous contamination within the diatom residue^69^. In each case, the Al concentrations were below 1%, and typically below 0.2%, indicating a high degree of diatom purity (Table S2). Samples were analysed for δ^18^O_diatom_ following a step-wise fluorination procedure developed by Leng *et al*.^70^, as summarised in Swann *et al*.^68^. The standard deviation (1σ) between repeat measurements of δ^18^O_diatom_ ranged from 0.05 to 0.4‰, with average of 0.23‰ (n = 9). Crucially, the magnitude of variations in δ^18^O_diatom_ are far greater than the analytical precision (Table S1).

## Supplementary information

Supplementary Information
