# Supplementary material for: Enhanced glacial discharge from the eastern Antarctic Peninsula since the 1700s associated with a positive Southern Annular Mode
Source: Sci Rep. 2019 Oct 24;9:14606. doi: 10.1038/s41598-019-50897-4 (PMC6813350; doi:10.1038/s41598-019-50897-4)
Supplement: Supplementary file 1 — Supplementary Information [file 41598_2019_50897_MOESM1_ESM.pdf]

Enhanced glacial discharge from the eastern Antarctic Peninsula since the 1700s associated with a positive Southern Annular Mode

Supplementary information

Dickens, W.A[1,2,5]., Kuhn, G[2]., Leng. M.J[3], Graham, A.G.C[4], Dowdeswell, J.A[5]., Meredith, M.P[1]., Hillenbrand, C.-D[1]., Hodgson, D.A[1]., Roberts, S.J[1]., Sloane, H[3]., Smith, J.A[1].

[1]British Antarctic Survey, High Cross, Madingley Road, Cambridge CB3 0ET, UK.
[2]Alfred-Wegener-Institut Helmholtz-Zentrum für Polar- und Meeresforschung, Bremerhaven. Germany.
[3]NERC Isotope Geosciences Laboratory, British Geological Survey, Keyworth, Nottingham, NG12 5GG, UK.
[4]College of Marine Science, University of South Florida, St Petersburg, Florida, USA.
[5]Scott Polar Research Institute, University of Cambridge, Cambridge CB2 1ER, UK.

Supplementary text

$\delta^{18}O_{diatom}$ as a proxy for glacier melt. Pike et al.[1] demonstrated that the $\delta^{18}O$ of marine diatoms ($\delta^{18}O_{diatom}$) provides a valuable proxy for reconstructing long term variations in glacial discharge[1]. As diatoms inhabit the upper portion of the water column, they are heavily influenced by the input of (glacial) meltwater from nearby terrestrial ice sheets[1-3]. The input of highly depleted glacial meltwater ($\delta^{18}O_{ice}$ = ~ 20‰[4]) reduces the $\delta^{18}O$ of the surrounding marine water. A lower $\delta^{18}O_{diatom}$ value is therefore indicative of an increase in glacial melting.

In a series of end-member mass balance calculations Swann et al.[2] were able to show that unrealistic changes in water source characteristics (outside of the meteoric water component) were required to shift the $\delta^{18}O_{water}$ beyond even the analytical (1σ) error in the $\delta^{18}O_{diatom}$ record. Given that the changes in the $\delta^{18}O_{diatom}$ in core PS67/182-1 are in excess of the 1σ error, water source changes (water mass and sea-ice melt) are considered unlikely. In contrast, just a 1.5% change in the volume of glacial melt would shift $\delta^{18}O_{water}$ beyond the analytical precision, highlighting water column sensitivity to glacial discharge. In addition, while SSTs will influence the $\delta^{18}O_{water}$, they only do so at 0.2‰ °C^{-1} [1]. There is no long-term SST record from the South Orkney region. However, given its close proximity to the western AP SST record[5], it seems unlikely that SSTs around the South Orkney Islands would have

substantially exceeded those from western AP; using the -0.2‰ °C^{-1}, correction, an ~18°C increase in SST would be required to drive the observed changes in PS67/182-1. Other secondary effects, such as silica maturation and species effects, are also considered to be negligible. We see a ~2 ‰ decrease in $\delta^{18}O_{diatom}$ within the upper ~100 cm of the core, opposite to that expected due to maturation, and consistent with other $\delta^{18}O_{diatom}$ studies that have ruled out silica maturation[3]. Furthermore, qualitative analysis of diatom abundance reveals an assemblage dominated by subgenus *Chaetoceros (Hyalochaete) spp.* resting spores*, Fragilariopsis curta* and *Thalassiosira antarctica'* but there is no significant correlation between changes in species composition/abundance and $\delta^{18}O_{diatom}$ values suggesting no significant species effects.

PS67/182-1 as an 'integrated' record of ice melt. Satellite measurements indicate that contemporary ice shelf thinning in the Weddell Sea embayment is concentrated along the eastern AP (Larsen B remnant, Larsen C and Larsen D ice shelves), whilst the Ronne-Filchner ice shelf shows minor thickening[6]. Despite limited data on ice shelf thickness change prior to the satellite era (1992-onwards) we anticipate that the spatial pattern of mass loss is similar to the present data, with the eastern AP dominating the discharge signal with a strong north-south gradient. The north-south gradient primarily reflects the position of the mean annual isotherm (-5°C), and is considered by some to be the theoretical limit of ice shelf viability[7]. Icebergs account for ~40-60% of contemporary mass loss in the region[8], and together with melt from basal melting of ice shelves, is entrained by the cyclonic, wind-driven Weddell Gyre before being ejected into the South Atlantic via the SOI and South Scotia Sea (Fig. 1a, c) (the so-called 'iceberg alley'[9]). Generally speaking, iceberg melt rates remain low until they reach warmer waters north of the Antarctic Polar Front where they ablate rapidly; an estimated 35% of giant icebergs' mass is exported north of 63°S (SOI is ~60°S) versus 3% for smaller bergs, although giant bergs spend more of the earlier part of their history nearer to the coast[10]. Whilst it is not possible to estimate a melt contribution from the South Orkney Island ice cap, the cumulative flow of meltwater from ice shelf and iceberg melting along the eastern AP is likely to be several orders of magnitude greater than any local input (from ice cap/glacier thinning). Indeed, some of the highest integrated freshwater fluxes – derived from ice shelf and iceberg melting - occur directly over our core site (Fig. 1a)[11,12]. We therefore favor a dominant eastern AP signal for our $\delta^{18}O_{diatom}$ record. We cannot, however, rule out a melt component from icebergs cavled from the wider Weddell Sea embayment or a melt component from the wider circum-Antarctic that is entrained within the coastal current and eventually within the Weddell Gyre.

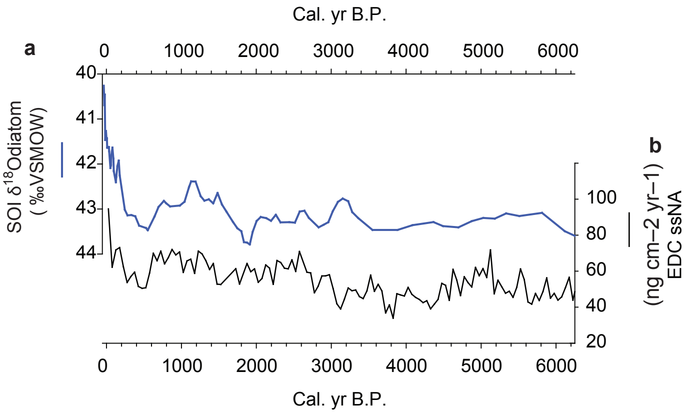

Figure S1. Glacial discharge versus an ice core wind proxy during the past 6,250 years. $\delta^{18}O_{diatom}$ glacial discharge (blue; 3 point moving average). **b**, EPICA Dome C sea salt aerosol flux (ssNa+) (black line)[13]. Higher ssNa+ values indicate stronger westerlies.

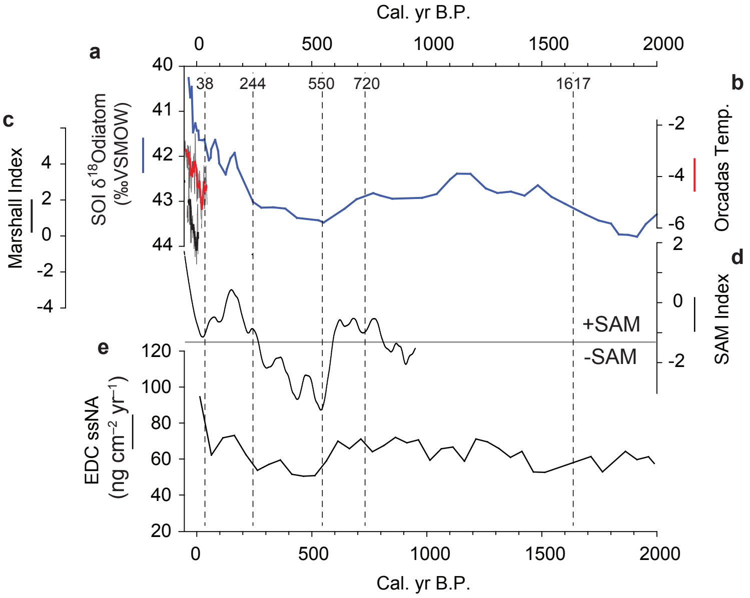

Figure S2. Glacial discharge versus records of Southern Annular Mode (SAM) and westerly winds during the past 2000 years. **a**, $\delta^{18}O_{diatom}$ glacial discharge (blue; 3 point moving average). **b**, Orcadas temperature record (Signy Island) (red line). **c**, Marshall Index of SAM[14]. **d**, SAM reconstruction[15]. Horizontal grey line denotes positive and negative SAM anomalies, above or below the long-term average (-1.3) (after[16]). **e**, EPICA Dome C ssNa+ aerosol flux[13]. Note the strong correlation between glacial discharge, atmospheric temperature and SAM. Dashed lines relate to change points in the $\delta^{18}O_{diatom}$ data discussed in the text.

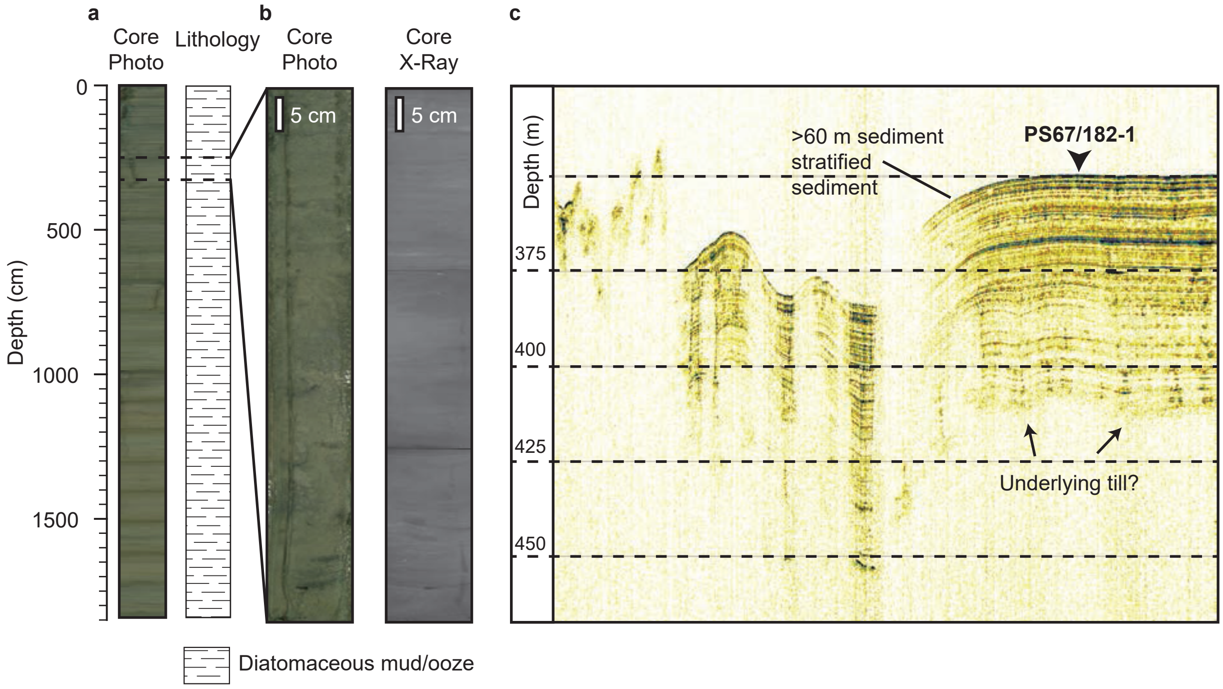

Figure S3. Core lithology and sub-bottom profile. **a**, Lithology and photo of core PS67/182-1. **b**, core photo and X-radiograph from 250-330 cm depth as a representative for the whole core. c, Parasound profile collected by the AWI during cruise ANT-XXII/4 showing the shallow seismic stratigraphy of core site PS67/182-1 - the core was recovered from a location with a thick (over 60 m) sedimentary infill.

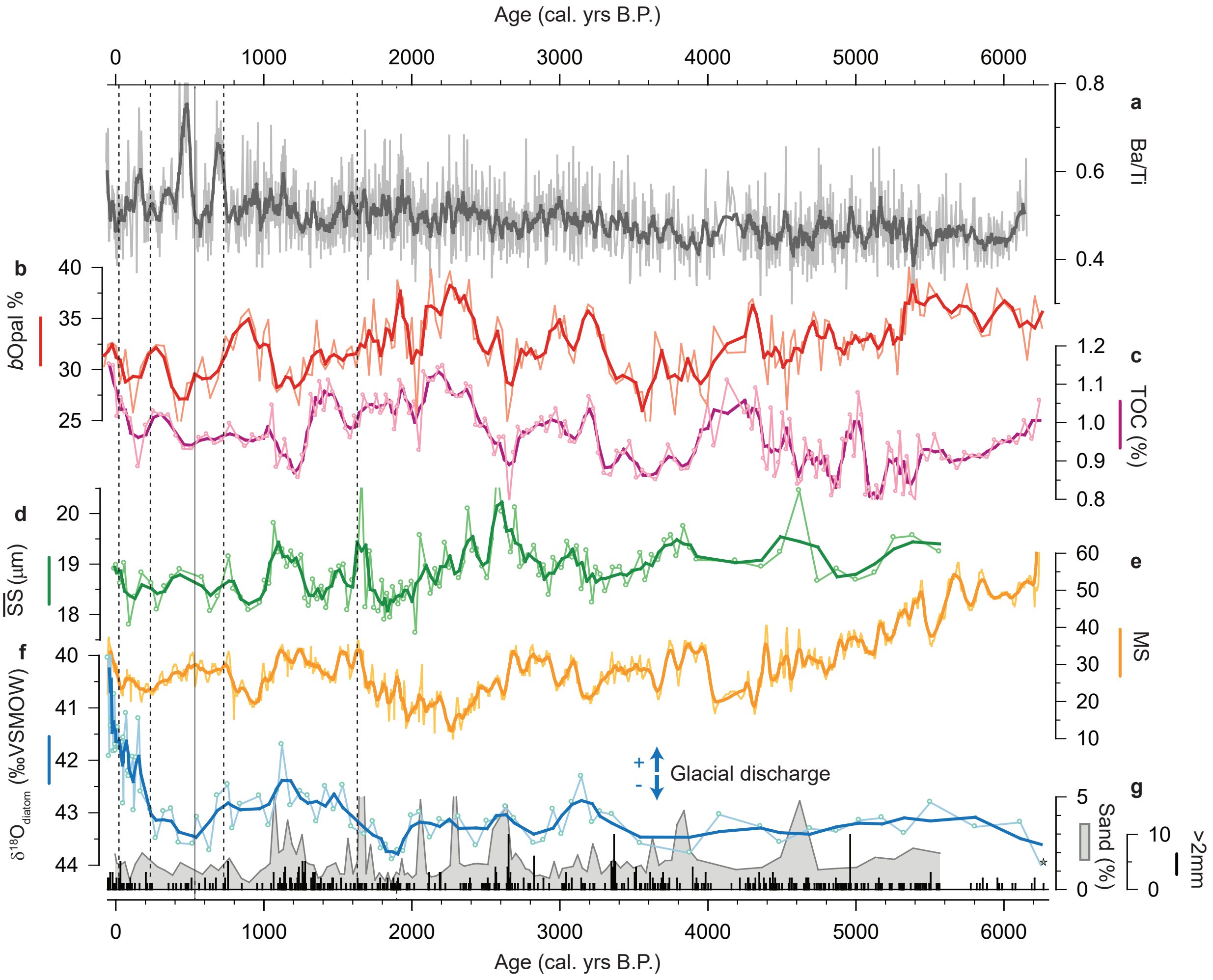

Figure S4. Multi-proxy data from PS67/182-1. **a**, Ba/Ti (black). **b**, biogenic opal % (*b*Opal; red). **c**, total organic carbon % (TOC; purple). **d**, mean sortable silt (SS; green). **e**, Magnetic susceptibility (MS; yellow). **f**, $\delta^{18}O_{diatom}$ glacial discharge (blue). All shown with a 3-point moving average (thick line). **g**, sand concentration (grey shaded), and number of clast counts > 2mm carried out at 2 cm core depth intervals on X-radiographs (black bars). The data are plotted against age (cal. yrs B.P.). Significant change points (Materials and Methods) are denoted by the vertical dotted lines.

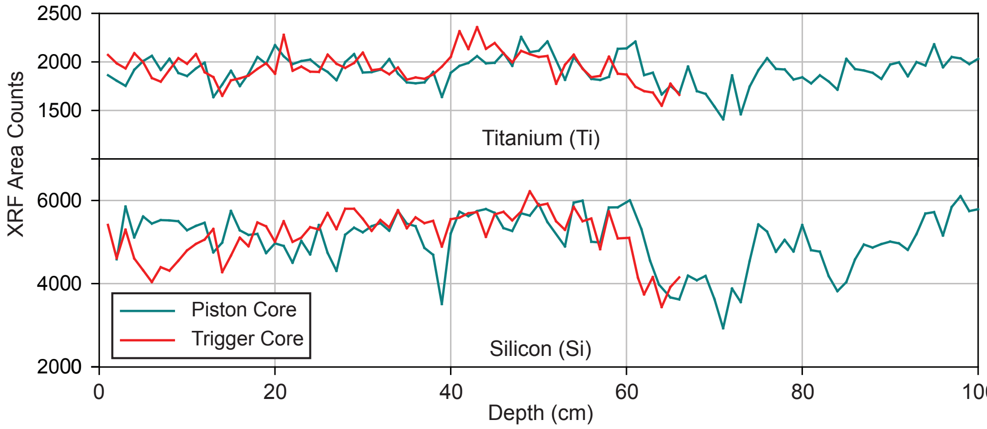

Figure S5. Piston and trigger core splice. Overlay of TC (red) onto PC (blue) to splice the cores together using XRF area counts of Titanium (Ti) and Silicon (Si). The plots indicate no vertical offset between the TC and PC, with a pronounced decrease in Si counts at around 60 cm in both cores.

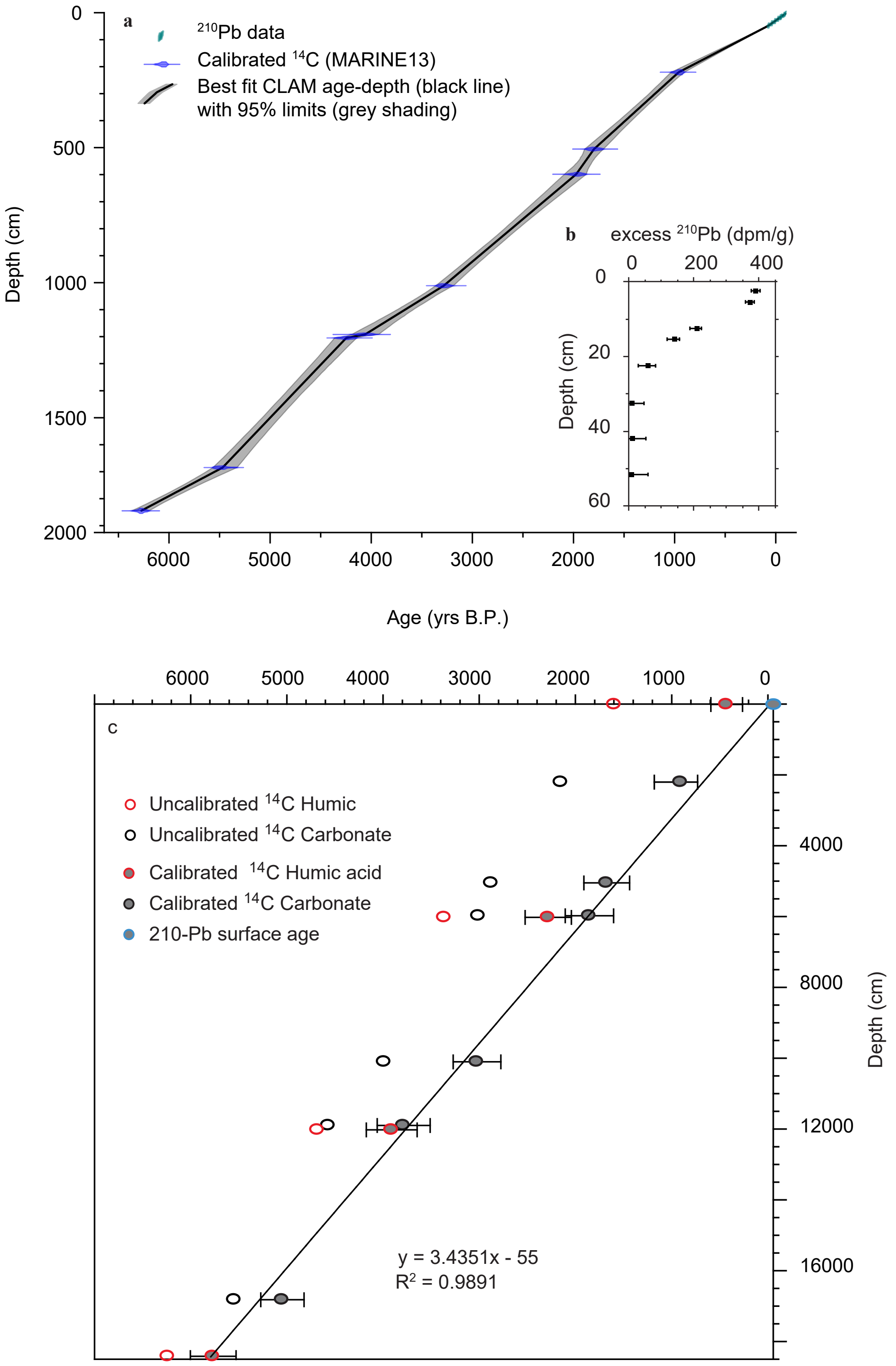

Figure S6. Age-depth model for core PS67/182-1. **a**, Stratigraphic age–depth plot undertaken in CLAM v2.2[17] using the Marine13 calibration curve[18]. The age model uses a linear interpolation between all points (^{14}C and ^{210}Pb), excluding the uppermost humic-acid age which is likely contaminated. The black line represents the 'best-fit' along with 95% confidence limits. Inset panel **b**, shows the down-core excess in the ^{210}Pb ($^{210}Pb_{xs}$ (Bq kg^{-1}) profile for the trigger core. The $^{210}Pb_{xs}$ concentration is 392.73 Bq kg^{-1} at the sediment surface and declines exponentially with depth. ^{137}Cs is below detection levels throughout. (c) Age-depth plot of ^{14}C data with a near-linear (R^2=0.9891) distribution of ages. Calibrated ages are shown as full black (carbonate), red (humic) and blue (modern surface from ^{210}Pb) circles with associated 2σ error bars.

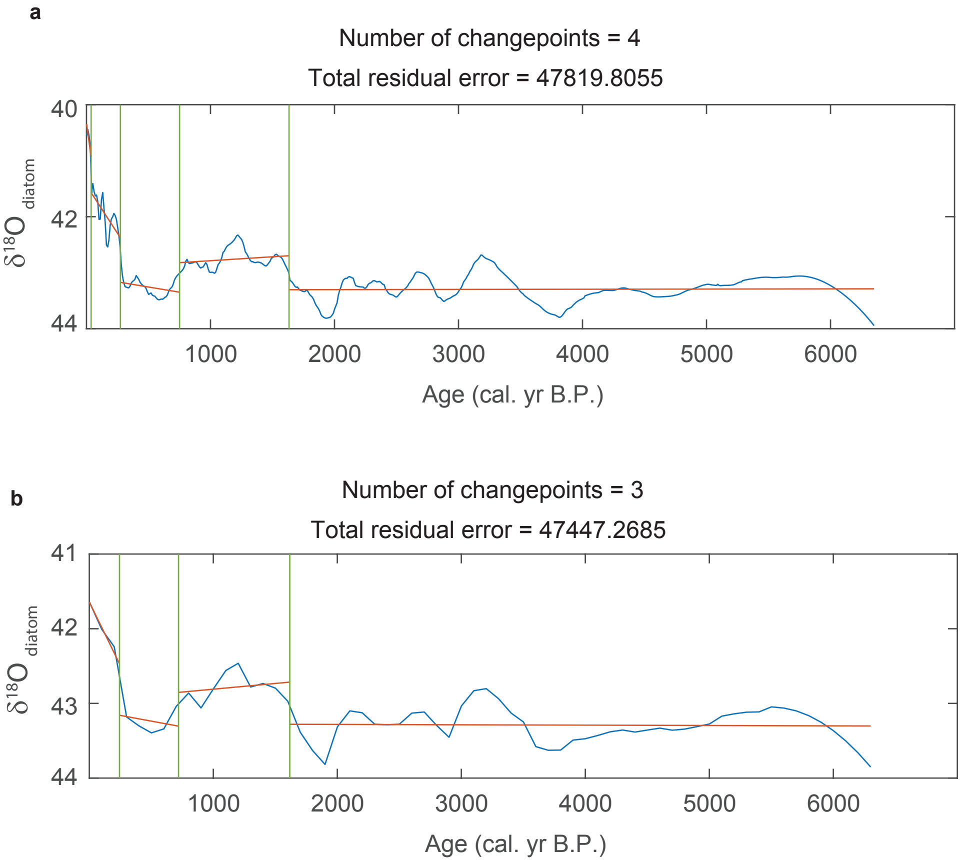

Figure S7. Change point analysis. **a**, 10 year LOESS-filtered data. **b**, 100 year LOESS-filtered data. Change points are consistent in both 10 and 100 year filtered datasets, although the 20th century change point (38 cal. yr BP) is missed in the 100-yr smoothing.

Supplementary table captions.

Table S1. $\delta^{18}O_{diatom}$ values obtained from core PS67/182-1 with corresponding depth together with the age and error (2σ) output from CLAM v2.2 (see Materials and Methods). TC = trigger core, PC = piston core.

Table S2. XRF results (in wt.%). SiO_2 percentages are between 94.64% and 99.99% and Al concentrations were below 1%, and typically below 0.2%, indicating a high degree of sample purity.

Table S3. Uncorrected and calibrated AMS ^{14}C dates from core PS67/182-1 on the SO continental shelf with sample depth and dated material (Humic = Humic acid, SF = Shell fragments, Bi. = Bivalve, Ga. = Gastropod, MB = Mixed benthic foraminifera). A marine reservoir effect (MRE) correction of 1300 yrs was applied. The 2σ range is given for each calibrated age (Min, Max) but the mean age is quoted for each sample. Calibration was undertaken in Calib v7.1 using the Marine13 curve[18]. AMS 14C dating was carried out at the NERC Radiocarbon Laboratory (Environment) in East Kilbride, UK; BETA Analytic Inc., Miami, Florida, U.S.A; ^{14}CHRONO Centre, Queen's University Belfast, UK and Leibniz-Labor, Kiel, Germany (with prefix SUERC, BETA, UBA and KIA).

Table S4. ^{210}Pb data for trigger core. The chronology is based on CRS modelling of the unsupported ^{210}Pb ($^{210}Pb_{xs}$) concentrations. Error bars denote ±1 s.d. of the ^{210}Pb.

Lab code	Corer	Depth (cm)	Age (cal. yrs B.P.)	error ± (yrs)	δ18Odiatom	Lab code	Corer	Depth (cm)	Age (cal. yrs B.P.)	error ± (yrs)	δ18Odiatom
FF31375	PC	4.25	-43	2	+40.05	FF31843	PC	420	1542	74	+42.48
FF31909	TC	7.5	-35	3	+41.93	FF31846	PC	440	1601	79	+43.27
FF31376	PC	10	-28	3	+38.80	FF31847	PC	470	1689	88	+43.42
FF31797	PC	12	-22	4	+41.35	FF31848, FF31852 [R]	PC	490	1747	96	+43.17
FF31910, FF31915 [R]	TC	14.25	-16	5	+41.21	FF31849	PC	510	1801	96	+43.69
FF31799	PC	18	-6	6	+41.84	FF31850	PC	528	1835	83	+43.62
FF31381	PC	20.5	1	7	+40.75	FF31851	PC	550	1876	74	+43.90
FF31800	PC	23	7	8	+41.71	FF31853	PC	570	1914	78	+43.72
FF31911	TC	24	10	7	+41.83	FF31854	PC	590	1952	98	+43.74
FF31386	PC	30	25	7	+41.38	FF31857	PC	610	2005	104	+43.06
FF31801	PC	33	33	8	+41.72	FF31859	PC	627.5	2060	97	+42.99
FF31912	TC	34.5	37	7	+41.77	FF31860, FF31866 [R]	PC	650	2133	94	+43.48
FF31380	PC	40	53	7	+41.67	FF31861	PC	670	2197	90	+43.13
FF31802	PC	43	61	7	+42.84	FF31863	PC	690	2261	86	+43.18
FF31916	TC	44	63	7	+41.58	FF31864	PC	710	2324	82	+43.08
FF31382, FF31388 [R]	PC	50	80	7	+41.11	FF31865	PC	747	2442	75	+43.63
FF31805	PC	53.5	93	8	+42.22	FF31867	PC	770	2516	73	+43.16
FF31917	TC	54.5	98	8	+42.31	FF31869	PC	790	2580	71	+43.11
FF31383	PC	60	126	9	+41.94	FF31870, FF31876 [R]	PC	810	2643	69	+42.89
FF31806	PC	61	131	9	+42.96	FF31872	PC	828	2701	68	+43.11
FF31918	TC	64	147	9	+41.96	FF31873	PC	869	2832	66	+43.59
FF31384	PC	67.5	165	10	+41.21	FF31874	PC	890	2899	67	+43.53
FF31807	PC	70	178	10	+42.60	FF31877	PC	910	2962	70	+42.94
FF31809	PC	83	245	14	+42.95	FF31878	PC	928.5	3021	78	+43.41
FF31810	PC	90	281	17	+43.50	FF31879	PC	950	3090	76	+42.80
FF31812	PC	100	333	20	+42.98	FF31881	PC	970	3154	79	+42.31
FF31813, FF31822 [R]	PC	110	385	24	+42.93	FF31882, FF31893 [R]	PC	993	3227	83	+43.19
FF31814	PC	120	436	28	+43.58	FF31883	PC	1010	3281	87	+42.99
FF31815	PC	138	529	35	+43.60	FF31885	PC	1028.5	3361	85	+43.04
FF31817	PC	149	586	39	+43.09	FF31886	PC	1072.5	3551	79	+43.59
FF31819	PC	160	643	44	+43.72	FF31887	PC	1150	3886	106	+43.77
FF31823	PC	170	695	48	+42.68	FF31889	PC	1192	4087	118	+43.04
FF31825	PC	184	767	53	+42.47	FF31890	PC	1250	4365	102	+43.27
FF31826	PC	190	798	55	+43.31	FF31894	PC	1300	4493	94	+43.57
FF31827	PC	200	850	60	+42.84	FF31895, FF31899 [R]	PC	1380	4698	89	+43.31
FF31828	PC	228	979	64	+42.68	FF31896	PC	1450	4877	87	+43.35
FF31830	PC	250	1043	57	+43.25	FF31898	PC	1510	5030	88	+43.15
FF31831	PC	270	1102	55	+42.59	FF31900	PC	1570	5184	95	+43.10
FF31833	PC	280	1131	56	+41.71	FF31902	PC	1628.5	5334	106	+43.39
FF31834	PC	300	1190	56	+42.87	FF31903	PC	1690	5511	111	+42.80
FF31835, FF31838 [R]	PC	323	1257	57	+42.60	FF31906	PC	1750	5814	77	+43.27
FF31836	PC	340	1307	60	+42.68	FF31907	PC	1810	6118	70	+43.19
FF31839	PC	360	1366	62	+43.17	FF31908	PC	1838	6259	85	+44.02
FF31840	PC	380	1425	66	+42.50						
FF31841	PC	400	1483	70	+42.95						

SM Table 1

Depth (cm)	MgO	Al2O3	SiO2	P2O5	SO3	K2O	CaO	TiO2	Mn3O4	Fe2O3	CO2
						%					
18	0.09	0.26	95	0.01	<0.01	0.03	0.03	<0.01	<0.01	0.05	4.88
33	0.07	0.18	99	<0.01	<0.01	<0.01	0.03	<0.01	<0.01	0.03	1.05
61	0.05	0.28	98	<0.01	<0.01	0.03	0.03	<0.01	<0.01	0.06	1.29
83	0.07	0.24	98	<0.01	0.02	0.02	0.03	<0.01	<0.01	0.05	1.76
110	0.07	0.16	97	<0.01	0.02	0.01	0.03	<0.01	<0.01	0.02	2.61
138	0.07	0.25	96	<0.01	0.03	0.02	0.03	<0.01	<0.01	0.05	3.06
160	0.06	0.19	95	0.01	0.03	0.02	0.03	<0.01	<0.01	0.05	4.24
184	0.06	0.26	97	<0.01	0.02	0.02	0.02	<0.01	<0.01	0.05	3.05
200	0.08	0.24	96	0.01	0.03	0.02	0.03	<0.01	<0.01	0.05	3.81
250	0.05	0.14	97	<0.01	0.03	0.01	0.03	<0.01	<0.01	0.02	3.08
300	0.12	0.12	97	<0.01	0.04	0.01	0.03	<0.01	<0.01	0.04	2.91
360	0.06	0.18	96	<0.01	0.02	0.02	0.03	<0.01	<0.01	0.03	3.81
400	0.09	0.22	96	0.01	0.03	0.02	0.03	<0.01	<0.01	0.03	3.94
470	0.09	0.16	95	0.01	0.03	0.02	0.02	<0.01	<0.01	0.03	4.60
510	0.06	0.19	96	<0.01	0.04	0.02	0.02	<0.01	<0.01	0.03	3.32
550	0.04	0.15	95	0.01	0.04	0.02	0.02	<0.01	<0.01	0.03	4.62
590	0.07	0.18	99	<0.01	0.05	0.01	0.03	<0.01	<0.01	0.03	0.97
627.5	0.02	0.17	99	<0.01	0.04	0.01	0.03	<0.01	<0.01	0.02	1.13
670	0.09	0.15	98	<0.01	0.04	0.02	0.03	<0.01	<0.01	0.03	1.60
710	0.06	0.19	99	<0.01	0.05	0.02	0.03	<0.01	<0.01	0.03	0.24
790	0.05	0.12	97	<0.01	0.06	0.01	0.03	<0.01	<0.01	0.03	2.94
828	0.03	0.18	97	<0.01	0.05	0.01	0.03	<0.01	<0.01	0.03	2.30
890	0.09	0.12	96	0.01	0.05	0.02	0.03	<0.01	<0.01	0.03	3.71
928.5	0.07	0.13	96	<0.01	0.05	0.02	0.03	<0.01	<0.01	0.03	3.41
970	0.07	0.15	98	<0.01	0.06	0.02	0.03	<0.01	<0.01	0.03	1.58
1010	0.07	0.16	97	<0.01	0.05	0.02	0.03	<0.01	<0.01	0.03	2.19
1072.5	0.04	0.16	98	<0.01	0.05	0.02	0.03	<0.01	<0.01	0.03	1.48
1192	0.06	0.15	96	0.01	0.04	0.02	0.03	<0.01	<0.01	0.03	3.91
1300	0.05	0.20	98	<0.01	0.05	0.02	0.03	<0.01	<0.01	0.03	1.87
1450	0.04	0.13	100	<0.01	0.06	0.02	0.02	<0.01	<0.01	0.03	<0.01
1628.5	0.04	0.19	98	<0.01	0.06	0.02	0.03	<0.01	<0.01	0.03	1.88
1810	0.08	0.19	100	<0.01	0.06	0.02	0.03	<0.01	<0.01	0.04	<0.01
7.5 TC	0.07	0.14	98	<0.01	<0.01	0.01	0.03	<0.01	<0.01	0.03	1.59
34.5 TC	0.09	0.16	97	<0.01	<0.01	0.01	0.02	<0.01	<0.01	0.03	2.21
64 TC	0.10	0.16	97	<0.01	<0.01	0.01	0.03	<0.01	<0.01	0.03	3.17

SM Table 2

Publication Code	Material dated	Depth (cm)	14C age (yrs B.P.)	± 1σ	MRE (ΔR)	Calibrated age (yrs B.P.)	± 2s	
							Min	Max
KIA 44018	Humic	1.5	1705	25	1300	**440**	257	616
UBA-25887	SF	220.5	2310	25	1300	**960**	730	1180
SUERC-54852	SF - Bi.	505	3099	37	1300	**1790**	1535	2052
SUERC-54853	SF - Ga.	598	3244	37	1300	**1990**	1717	2263
BETA-407308	SF	1010.25	4310	30	1300	**3260**	2992	3532
SUERC-54434	Foram - MB	1190	4942	42	1300	**4090**	3793	4393
KIA 44020	Humic	1202.5	5065	30	1300	**4230**	3940	4515
SUERC-54435	Foram - MB	1682	6008	42	1300	**5470**	5221	5711
KIA 44021	Humic	1841.75	6760	35	1300	**6250**	6002	6497

SM Table 3

Supplementary References

1 Pike, J., Swann, G. E. A., Leng, M. J. & Snelling, A. M. Glacial discharge along the west Antarctic Peninsula during the Holocene. *Nature Geoscience* 6, 199-202, doi:10.1038/ngeo1703 (2013).

2 Swann, G. E. A., Pike, J., Snelling, A. M., Leng, M. J. & Williams, M. C. Seasonally resolved diatom delta O-18 records from the West Antarctic Peninsula over the last deglaciation. *Earth Planet. Sci. Lett.* 364, 12-23, doi:10.1016/j.epsl.2012.12.016 (2013).

3 Crespin, J. *et al.* Holocene glacial discharge fluctuations and recent instability in East Antarctica. *Earth Planet. Sci. Lett.* 394, 38-47, doi:10.1016/j.epsl.2014.03.009 (2014).

4 Meredith, M. P. *et al.* Changes in the freshwater composition of the upper ocean west of the Antarctic Peninsula during the first decade of the 21st century. *Progress in Oceanography* 87, 127-143, doi:10.1016/j.pocean.2010.09.019 (2010).

5 Shevenell, A. E., Ingalls, A. E., Domack, E. W. & Kelly, C. Holocene Southern Ocean surface temperature variability west of the Antarctic Peninsula. *Nature* 470, 250-254, doi:10.1038/nature09751 (2011).

6 Paolo, F. S., Fricker, H. A. & Padman, L. Volume loss from Antarctic ice shelves is accelerating. *Science* 348, 327-331, doi:10.1126/science.aaa0940 (2015).

7 Morris, E. M. & Vaughan, D. G. in *Antarctic Peninsula Climate Variability* Vol. 79 (eds E. Domack *et al.*) 61-69 (AGU, Antarctic Research Series., Washington DC, 2003).

8 Depoorter, M. A. *et al.* Calving fluxes and basal melt rates of Antarctic ice shelves (vol 502, pg 89, 2013). *Nature* 502, doi:10.1038/nature12737 (2013).

9 Weber, M. E. *et al.* Millennial-scale variability in Antarctic ice-sheet discharge during the last deglaciation. *Nature* 510, 134-+, doi:10.1038/nature13397 (2014).

10 Silva, T. A. M., Bigg, G. R. & Nicholls, K. W. Contribution of giant icebergs to the Southern Ocean freshwater flux. *J. Geophys. Res.-Oceans* 111, doi:10.1029/2004jc002843 (2006).

11 Merino, N. *et al.* Antarctic icebergs melt over the Southern Ocean: Climatology and impact on sea ice. *Ocean Modelling* 104, 99-110, doi:10.1016/j.ocemod.2016.05.001 (2016).

12 Tournadre, J., Bouhier, N., Girard-Ardhuin, F. & Remy, F. Antarctic icebergs distributions 1992-2014. *J. Geophys. Res.-Oceans* 121, 327-349, doi:10.1002/2015jc011178 (2016).

13 Rothlisberger, R. *et al.* Limited dechlorination of sea-salt aerosols during the last glacial period: Evidence from the European Project for Ice Coring in Antarctica (EPICA) Dome C ice core. *J. Geophys. Res.-Atmos.* 108, art. no.-4526 (2003).

14 Marshall, G. J. Trends in the southern annular mode from observations and reanalyses. *J. Clim.* 16, 4134-4143, doi:10.1175/1520-0442(2003)016<4134:titsam>2.0.co;2 (2003).

15 Abram, N. J. *et al.* Evolution of the Southern Annular Mode during the past millennium. *Nature Climate Change* 4, 564-569, doi:10.1038/nclimate2235 (2014).

16 Bertler, N. A. N. *et al.* The Ross Sea Dipole – temperature, snow accumulation and sea ice variability in the Ross Sea region, Antarctica, over the past 2700 years. *Clim. Past* 14, 193-214, doi:10.5194/cp-14-193-2018 (2018).

17 Blaauw, M. Methods and code for 'classical' age-modelling of radiocarbon sequences. *Quaternary Geochronology* 5, 512-518, doi:10.1016/j.quageo.2010.01.002 (2010).

18 Reimer, P. J. *et al.* INTCAL13 AND MARINE13 RADIOCARBON AGE CALIBRATION CURVES 0-50,000 YEARS CAL BP. *Radiocarbon* 55, 1869-1887, doi:10.2458/azu_js_rc.55.16947 (2013).
